# Risk factors for BK virus infection in DCD donor kidney transplant recipients

**DOI:** 10.3389/fmed.2023.1181743

**Published:** 2023-07-12

**Authors:** Yiting Liu, Chenyang Kong, Haochong Hu, Yalong Zhang, Tianyu Wang, Tao Qiu, Jiangqiao Zhou

**Affiliations:** ^1^Department of Organ Transplantation, Renmin Hospital of Wuhan University, Wuhan, Hubei, China; ^2^Department of Urology, Renmin Hospital of Wuhan University, Wuhan, Hubei, China

**Keywords:** kidney transplantation, BK viruria, BK viremia, risk factors, donation after cardiac death, machine learning

## Abstract

**Background:**

BK virus infection after kidney transplantation can negatively impact the prognosis of patients. However, current risk factor analyses primarily focus on BK virus nephropathy, while BK viruria and BK viruria progressing to BK viremia receive less attention. This study aims to analyze the risk factors associated with BK viruria and BK viruria progressing to BK viremia in recipients of donation after cardiac death (DCD), with the goal of facilitating early intervention.

**Methods:**

Donor characteristics and clinical data of recipients before and after transplantation were evaluated, and logistic univariate and multivariate analyses were performed to determine the risk factors associated with BK viruria and the progression of BK viruria to BK viremia. Additionally, machine learning techniques were employed to identify the top five features associated with BK viruria evolving into BK viremia.

**Results:**

During a median follow-up time of 1,072 days (range 739–1,418), 69 transplant recipients (15.6% incidence rate) developed BK viruria after transplantation, with 49.3% of cases occurring within 6 months post-transplantation. Moreover, 19 patients progressed to BK viremia. Donor age [OR: 1.022 (1.000, 1.045), *p* = 0.047] and donor procalcitonin (PCT) levels [0.5–10 ng/ml; OR: 0.482 (0.280, 0.828), *p* = 0.008] were identified as independent risk factors for BK viruria. High BK viruria [OR: 11.641 (1.745, 77.678), *p* = 0.011], recipient age [OR: 1.106 (1.017, 1.202), *p* = 0.018], and immunoinduction regimen [ATG; OR: 0.063 (0.006, 0.683), *p* = 0.023] were independent risk factors for BK viruria progressing to BK viremia. Machine learning analysis confirmed the importance of high BK viruria, recipient age, and immunoinduction regimen (ATG) in predicting the progression of BK viruria to BK viremia.

**Conclusion:**

The development and progression of BK virus in DCD kidney transplant recipients is influenced by multiple factors. Early intervention and treatment could potentially extend the lifespan of the transplanted organ.

## Background

The BK virus is a common resident in healthy individuals and typically reactivates when the immune system is weakened (1, 2). After a BK virus infection, kidney transplant patients commonly develop BK viruria, which may later progress to BK viremia and BKVN (3–6). BKVN is a significant cause of graft failure, affecting up to 10% of kidney transplant recipients and resulting in graft loss in up to 50% of those affected (7). Presently, there is no effective treatment plan for BK virus infection, and management primarily involves reducing immunosuppressive dosages and relying on autocellular immunity to achieve antiviral effects (8).

The screening of risk factors for BK virus infection mainly focuses on BKVN and BK viremia. A meta-analysis has summarized the risk factors for BK viremia and BKVN (9). It has been found that deceased donors are an independent risk factor for BK viremia. However, there are limited studies on the risk factors of BK viruria and BK viruria evolving into BK viremia in the context of DCD (10). BK viruria has been reported to progress to BK viremia in around 33% of cases, and high levels of BK viruria can be employed as a screening tool for BK viremia and BKVN in kidney transplant recipients (11). Moreover, persistent BK viruria can be used as an early marker of BKVN development (12). Therefore, early detection of BK viruria and clinical intervention for those at a high risk of BK viruria progressing to BK viremia are essential to control the progression of the disease as much as possible.

Traditional logistic regression has long been the primary method for investigating risk factors associated with BK virus infection (13, 14). However, machine learning techniques have shown promise in predicting complex clinical data (15). For example, recent research has applied machine learning to proteomic analysis of extracellular vesicles to identify potential biomarkers of BK viruria and BK viremia (16). However, there are no studies that have yet used machine learning to explore the risk factors associated with BK virus infection using clinical data of donors and recipients. Therefore, this study aims to conduct a retrospective study at a single center. Specifically, the study will first employ traditional logistic regression to identify potential risk factors for BK viruria and BK viruria evolving into BK viremia. Thereafter, a random forest model will be used to determine the importance ranking of potential risk factors for developing BK viremia, as a validation of the potential risk factors identified by traditional logistic regression.

## Materials and methods

### Patient groups

**Figure 1:**
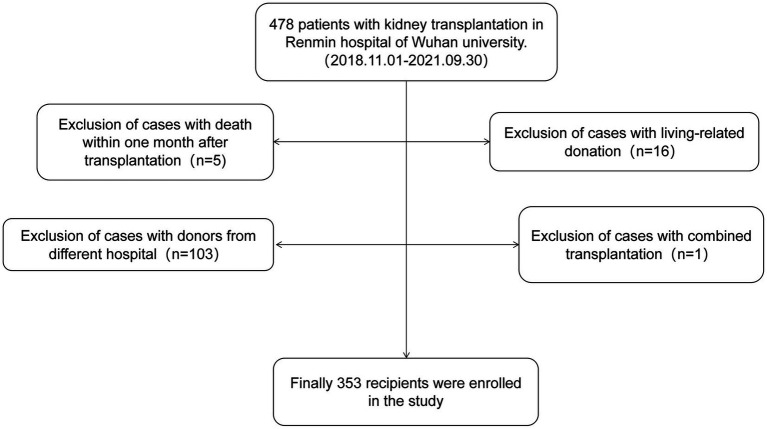
Flowchart of patient selection in the present study.

This study enrolled 353 kidney transplant recipients who received kidneys from deceased donors in the Organ Transplantation Department of Renmin Hospital of Wuhan University from November 2018 to September 2021. Inclusion criteria required single kidney transplantation from organ donation after cardiac death (DCD), and all DCD donors were Maastricht III. Exclusion criteria included allocation of donor kidneys from other hospitals, combined heart-kidney or liver-kidney transplantation, death within 1 month after surgery and living kidney transplantation. The flow chart of case screening is shown in Figure 1. Recipients were followed up until May 2023. The study was approved by the Ethics Committee of Renmin Hospital of Wuhan University. The kidney transplant patients were classified into two groups: the control group and the BK viruria group. It is important to note that all patients with BK viremia had previously developed BK viruria. As a result, the BK viruria group was further divided into a control group and a BK viremia group based on whether or not BK viruria had progressed to BK viremia.

### BKV surveillance protocol

In order to track BK virus (BKV) DNA levels in urine and blood, we employed quantitative polymerase chain reaction (qPCR) at regular intervals. Specifically, we conducted monthly monitoring in the first year post-transplant, every 3 months in the second year, and annually thereafter until the fifth year.

### Diagnostic criteria

The diagnostic criteria for acute kidney injury (AKI) followed the guidelines recommended by the Kidney Disease: Improving Global Outcomes (KDIGO), which included an increase in serum creatinine (sCr) level ≥ 26.5 µmol/l (0.3 mg/dl) within 48 h or a known or presumed increase in sCr to 1.5 times or more of the baseline value within the past 7 days (17). Delayed graft function (DGF) was defined as the need for dialysis within 1 week after kidney transplantation (18). Due to the lower limit of quantitation was 10^3^ copies/ml. BK viruria was defined as the detection of BKV DNA load in urine ≥10^3^ copies /ml, and high BK viruria was defined as the detection of BKV DNA load in urine ≥10^7^ copies/ml (19). BK viremia was defined as the detection of BKV DNA load in blood ≥10^3^ copies/ml (20).

### Data collection

The collection of clinical data involves both donor pre-donation data and recipient clinical data. Donor data include: (1) Clinical data such as gender, age, BMI and blood group; (2) Comorbidities and primary disease; (3) Relevant laboratory test indicators such as terminal albumin, terminal urea, terminal serum creatinine, terminal eGFR, terminal hemoglobin, terminal urine protein, terminal procalcitonin (PCT), and terminal hematuria sputum culture. Recipient data included: (1) Clinical data such as gender, age, BMI, and blood group; (2) Type matching data including PRA, HLA mismatch number; (3) Dialysis data including preoperative dialysis mode and duration; (4) Comorbidities and primary disease; (5) Postoperative laboratory examination indicators such as BKV DNA load in urine, BK viruria time, BKV DNA load in blood, and BK viremia time; (6) Hospitalization information including length of hospitalization (LOH) and DGF; (7) Immunosuppressant use such as Immunosuppressive and induction regimen.

### Statistical analysis

The statistical analysis was performed utilizing the Statistical Package for Social Sciences (SPSS), version 25.0 (SPSS Inc., Chicago, IL, USA), and R4.2.1. The continuous variables were determined using either an independent t-test or Mann–Whitney U test and presented as means ± SD or medians with interquartile ranges, respectively. Categorical variables were evaluated using the Chi-squared or Fisher’s exact tests and expressed as numbers (percentages). Initially, a univariate logistic proportional risk regression model was fitted for BK viruria in the entire renal transplant population. Subsequently, variables with *p* < 0.1 were included in a multivariate logistic proportional risk regression model. In addition, a single-factor logistic proportional risk regression model for BK viremia was fitted for the population with BK viruria, and variables with *p* < 0.1 were included in a multivariate logistic proportional risk regression model. All statistical tests and confidence intervals were two-sided, and *p* < 0.05 were considered to be statistically significant. To validate the results obtained through logistic regression, a machine learning approach was used to assess the importance of potential risk factors for the progression of BK viruria to BK viremia. Categorical variables were treated in the dataset of 69 BK viruria cases by creating dummy variables, and the number of variables was reduced by lasso regression and 10-fold cross-validation, with the final number of variables determined by the lambda with the minimum mean square error. We used the random forest model for model fitting, with the dependent variable set to whether the patient progressed to BK viremia and the independent variables set to features other than the dependent variable, ranked in importance using Gini importance for the independent variables.

## Results

### Comparison of donor and recipient data between BK viruria group and control group

This study analyzed a total of 353 kidney transplant recipients, of which 284 were free of BK virus infection, and 69 had BK viruria after the transplant surgery. All donors provided DCD kidneys, and the transplantation was carried out using a single kidney from a donor with the same blood group as that of the recipient. The immunosuppressive maintenance regimen used by 97.7% of the recipients was Tacrolimus + Mycophenolate mofetil + Glucocorticoid (TAC + MMF + GC), and 37.1% of the recipients received Antihuman thymocyte globulin (ATG) for immune induction. When compared to the control group, the BK viruria group had a higher donor age (58.00 [47.00, 63.00] vs. 53.00 [44.00, 60.00], *p* = 0.01) and a greater proportion of donor pre-donor PCT at 0.5-10 ng/ml (40.6 vs. 58.1%, *p* = 0.013). No statistical differences were observed in other comparisons. Please refer to Table 1 for detailed data.

**Table 1:** Demographics and clinical characteristics of donors (kidneys) and recipients.

Characteristic	All, *n* = 353	Control, *n* = 284	BK viruria, *n* = 69	*p*-value
Donor (kidneys) characteristic				
Age, year	54.00 [45.00, 61.00]	53.00 [44.00, 60.00]	58.00 [47.00, 63.00]	0.01
Male, *n*	304 (86.1)	245 (86.3)	59 (85.5)	1
Blood group, *n*				0.791
A	124 (35.1)	103 (36.3)	21 (30.4)	
B	75 (21.2)	58 (20.4)	17 (24.6)	
AB	34 (9.6)	27 (9.5)	7 (10.1)	
O	120 (34.0)	96 (33.8)	24 (34.8)	
BMI, kg/m^2^	22.86 [20.76, 24.49]	22.86 [20.96, 24.49]	22.49 [20.76, 25.35]	0.651
Cause of death, *n*				0.357
Cerebral hemorrhage	165 (46.7)	126 (44.4)	39 (56.5)	
Cerebral infarction	94 (26.6)	78 (27.5)	16 (23.2)	
Cerebral trauma	32 (9.1)	29 (10.2)	3 (4.3)	
Brain tumor	33 (9.3)	27 (9.5)	6 (8.7)	
Others	29 (8.2)	24 (8.5)	5 (7.2)	
Hypertension, *n*	149 (42.2)	115 (40.5)	34 (49.3)	0.234
Diabetes, *n*	28 (7.9)	19 (6.7)	9 (13.0)	0.133
Al, g/L	35.60 [33.42, 38.20]	35.60 [34.00, 38.31]	35.30 [32.80, 37.30]	0.151
Urea, mmol/l	8.60 [6.15, 13.42]	8.20 [6.01, 13.31]	10.30 [6.48, 13.42]	0.127
sCr, μmol/l	59.00 [41.00, 83.00]	58.00 [40.00, 83.75]	60.00 [45.00, 79.00]	0.784
eGFR, ml/min	108.24 [87.24, 129.00]	111.22 [82.20, 131.20]	101.06 [92.00, 114.66]	0.141
Hemoglobin, g/l	106.00 [95.00, 123.00]	106.00 [95.00, 124.00]	106.00 [95.00, 121.00]	0.798
Urine protein, n	134 (38.0)	111 (39.1)	23 (33.3)	0.456
AKI, *n*	50 (14.2)	43 (15.1)	7 (10.1)	0.382
PCT, ng/ml				0.013
<0.5 or >10	160 (45.3)	119 (41.9)	41 (59.4)	
0.5–10	193 (54.7)	165 (58.1)	28 (40.6)	
Culture, n	181 (51.3)	145 (51.1)	36 (52.2)	0.974
Recipient characteristic				
Age, year	42.76 ± 10.51	42.77 ± 10.59	42.74 ± 10.25	0.982
Male, *n*	251 (71.1)	205 (72.2)	46 (66.7)	0.448
Blood group, *n*				0.88
A	120 (34.0)	99 (34.9)	21 (30.4)	
B	78 (22.1)	61 (21.5)	17 (24.6)	
AB	38 (10.8)	31 (10.9)	7 (10.1)	
O	117 (33.1)	93 (32.7)	24 (34.8)	
BMI, kg/m^2^	21.48 [19.23, 23.67]	21.48 [19.51, 23.66]	21.61 [18.67, 23.92]	0.625
Dialysis modalities, *n*				0.702
No	33 (9.3)	25 (8.8)	8 (11.6)	
Hematodialysis	59 (16.7)	47 (16.5)	12 (17.4)	
Peritoneal dialysis	255 (72.2)	208 (73.2)	47 (68.1)	
Both	6 (1.7)	4 (1.4)	2 (2.9)	
Dialysis time, mth	12.00 [5.00, 28.00]	12.00 [6.00, 30.00]	8.00 [3.00, 24.00]	0.06
Hypertension, *n*	319 (90.4)	258 (90.8)	61 (88.4)	0.698
Diabetes, *n*	25 (7.1)	21 (7.4)	4 (5.8)	0.84
Hepatitis B, *n*	58 (16.4)	46 (16.2)	12 (17.4)	0.953
Transplantation etiology, *n*				0.317
Chronic glomerulonephritis	265 (75.1)	213 (75.0)	52 (75.4)	
Hypertensive nephrosclerosis	16 (4.5)	15 (5.3)	1 (1.4)	
Polycystic kidney disease	14 (4.0)	13 (4.6)	1 (1.4)	
Diabetic nephropathy	17 (4.8)	13 (4.6)	4 (5.8)	
Others	41 (11.6)	30 (10.6)	11 (15.9)	
HLAmm, *n*	4.00 [3.00, 5.00]	4.00 [3.00, 5.00]	4.00 [3.00, 5.00]	0.922
PRA, *n*				1
<10%	257 (72.8)	207 (72.9)	50 (72.5)	
≥10%	96 (27.2)	77 (27.1)	19 (27.5)	
DGF, *n*	46 (13.0)	38 (13.4)	8 (11.6)	0.845
LOH, d	20.00 [18.00, 23.00]	20.00 [18.00, 23.00]	20.00 [19.00, 22.00]	0.433
Immunosuppressive regimen, *n*				1
CsA + MMF + GC	8 (2.3)	6 (2.1)	2 (2.9)	
TAC + MMF + GC	345 (97.7)	278 (97.9)	67 (97.1)	
Immunoinduction regimen, n				0.254
Basiliximab	222 (62.9)	174 (61.3)	48 (69.6)	
ATG	131 (37.1)	110 (38.7)	21 (30.4)	

BMI, body mass index; Al, terminal Albumin; Ur, terminal Urea; sCr, terminal serum creatinine; eGFR, terminal estimated glomerular filtration rate; Hemoglobin, terminal Hemoglobin; Urine protein, terminal Urine protein was present; AKI, Acute Kidney Injury; PCT, procalcitonin; Culture, terminal hematuria sputum culture status any one is positive; PRA, panel reactive antibodies; HLA mm, HLA mismatch; DGF: Delayed Graft Function; LOH: The length of post-kidney transplant hospitalization; ATG, Antihuman thymocyte globulin; TAC, Tacrolimus; CsA, Cyclosporin A; MMF, Mycophenolate mofetil; GC, Glucocorticoid.

### Analysis of risk factors for BK viruria

The association between BK viruria and various variables was analyzed using a univariate logistic proportional risk regression model. Variables with a value of p less than 0.1 were included in the multivariate logistic proportional risk regression model. The univariate analysis identified certain variables with *p* < 0.1, such as donor age, donor diabetes (yes), and donor PCT (0.5–10 ng/ml). After including these variables in the multivariate analysis, it was found that donor age [OR: 1.022 (1.000, 1.045), *p* = 0.047] and donor PCT [0.5–10 ng/ml; OR: 0.482 (0.280, 0.828), *p* = 0.008] were independent predictors of BK viruria. Table 2 provides detailed information about the analysis.

**Table 2:** Univariate and multivariate analysis of BK viruria.

Variable	Single-factor analysis, OR (95% CI)	*p*-value	Multiple-factor analysis, OR (95% CI)	*p*-value
Donor age, year	1.023 (1.001,1.046)	0.037	1.022 (1.000,1.045)	0.047
Donor diabetes(yes)	2.092 (0.902,4.852)	0.085		
Donor PCT (0.5 ~ 10 ng/ml)	0.493 (0.288,0.841)	0.01	0.482 (0.280,0.828)	0.008

OR, odds ratio; 95% CI, 95% confidence interval; PCT: procalcitonin.

### Comparison of donor and recipient data between BK viremia group and control group

After stratifying the 69 patients with BK viruria into control and BK viremia groups based on whether they developed BK viremia or not, we observed that the BK viremia group had a higher recipient age (47.68 ± 7.34 vs. 40.86 ± 10.63, *p* = 0.012), a higher proportion of high BK viruria (89.5 vs. 54.0%, *p* = 0.014), and a lower proportion of ATG use (5.3 vs. 40.0%, *p* = 0.012), as compared to the control group. However, no significant differences were observed in the remaining donor-recipient profiles, and detailed donor-recipient profiles can be found in Table 3.

**Table 3:** Demographic and clinical characteristics of donor (kidney) and recipient of BK viruria evolving into BK viremia.

Characteristic	BK viruria, *n* = 69	Control, *n* = 50	BK viremia, *n* = 19	*p*-value
Donor (kidneys) characteristic				
Age, year	58.00 [47.00, 63.00]	56.00 [45.00, 63.75]	62.00 [52.00, 63.00]	0.211
Male, *n*	59 (85.5)	44 (88.0)	15 (78.9)	0.568
Blood group				0.107
A	21 (30.4)	13 (26.0)	8 (42.1)	
B	17 (24.6)	10 (20.0)	7 (36.8)	
AB	7 (10.1)	6 (12.0)	1 (5.3)	
O	24 (34.8)	21 (42.0)	3 (15.8)	
BMI, kg/m^2^	22.87 ± 3.42	22.77 ± 3.56	23.13 ± 3.10	0.698
Cause of death, *n*				0.643
Cerebral hemorrhage	39 (56.5)	27 (54.0)	12 (63.2)	
Cerebral infarction	16 (23.2)	11 (22.0)	5 (26.3)	
Cerebral trauma	3 (4.3)	2 (4.0)	1 (5.3)	
Brain tumor	6 (8.7)	6 (12.0)	0 (0.0)	
Others	5 (7.2)	4 (8.0)	1 (5.3)	
Hypertension, *n*	34 (49.3)	23 (46.0)	11 (57.9)	0.54
Diabetes, *n*	9 (13.0)	4 (8.0)	5 (26.3)	0.106
Al, g/l	35.25 ± 3.61	35.16 ± 3.99	35.50 ± 2.42	0.736
Urea, mmol/l	10.30 [6.48, 13.42]	10.30 [6.55, 14.04]	10.30 [6.32, 11.76]	0.677
sCr, μmol/l	60.00 [45.00, 79.00]	62.00 [45.50, 82.00]	60.00 [45.50, 72.00]	0.648
eGFR, ml/min	101.06 [92.00, 114.66]	100.81 [91.76, 114.91]	106.50 [97.72, 110.61]	0.424
Hemoglobin, g/l	106.00 [95.00, 121.00]	105.50 [95.00, 119.25]	106.00 [97.00, 121.50]	0.364
Urine protein, *n*	46 (66.7)	34 (68.0)	12 (63.2)	0.924
AKI, n	7 (10.1)	7 (14.0)	0 (0.0)	0.203
PCT, ng/ml				0.665
<0.5 or>10	41 (59.4)	31 (62.0)	10 (52.6)	
0.5–10	28 (40.6)	19 (38.0)	9 (47.4)	
Culture, *n*	36 (52.2)	23 (46.0)	13 (68.4)	0.163
Recipient characteristic				
Age, year	42.74 ± 10.25	40.86 ± 10.63	47.68 ± 7.34	0.012
Male, *n*	46 (66.7)	33 (66.0)	13 (68.4)	1
Blood group, *n*				0.107
A	21 (30.4)	13 (26.0)	8 (42.1)	
B	17 (24.6)	10 (20.0)	7 (36.8)	
AB	7 (10.1)	6 (12.0)	1 (5.3)	
O	24 (34.8)	21 (42.0)	3 (15.8)	
BMI, kg/m^2^	21.38 ± 3.31	21.39 ± 3.40	21.36 ± 3.12	0.978
Dialysis modalities, *n*				0.415
No	8 (11.6)	5 (10.0)	3 (15.8)	
Hematodialysis	12 (17.4)	7 (14.0)	5 (26.3)	
Peritoneal dialysis	47 (68.1)	36 (72.0)	11 (57.9)	
Both	2 (2.9)	2 (4.0)	0 (0.0)	
Dialysis time, mth	8.00 [3.00, 24.00]	7.50 [3.25, 18.25]	12.00 [4.00, 24.00]	0.505
Hypertension, *n*	61 (88.4)	45 (90.0)	16 (84.2)	0.803
Diabetes, *n*	4 (5.8)	4 (8.0)	0 (0.0)	0.488
Hepatitis B, *n*	12 (17.4)	9 (18.0)	3 (15.8)	1
Transplantation etiology, *n*				0.127
Chronic glomerulonephritis	52 (75.4)	35 (70.0)	17 (89.5)	
Hypertensive nephrosclerosis	1 (1.4)	1 (2.0)	0 (0.0)	
Polycystic kidney disease	1 (1.4)	0 (0.0)	1 (5.3)	
Diabetic nephropathy	4 (5.8)	4 (8.0)	0 (0.0)	
Others	11 (15.9)	10 (20.0)	1 (5.3)	
HLAmm, *n*	4.00 [3.00, 5.00]	4.00 [3.00, 5.00]	4.00 [3.00, 4.00]	0.572
PRA, *n*				1
<10%	50 (72.5)	36 (72.0)	14 (73.7)	
≥10%	19 (27.5)	14 (28.0)	5 (26.3)	
DGF, *n*	8 (11.6)	8 (16.0)	0 (0.0)	0.152
LOH, d	20.00 [19.00, 22.00]	20.00 [19.00, 22.00]	19.00 [18.00, 20.00]	0.2
Immunosuppressive regimen, *n*				0.478
CsA + MMF + GC	2 (2.9)	1 (2.0)	1 (5.3)	
TAC + MMF + GC	67 (97.1)	49 (98.0)	18 (94.7)	
Immunoinduction regimen, *n*				0.012
Basiliximab	48 (69.6)	30 (60.0)	18 (94.7)	
ATG	21 (30.4)	20 (40.0)	1 (5.3)	
High BK viruria	44 (63.8)	27 (54.0)	17 (89.5)	0.014

BMI, Body Mass Index; Al, terminal Albumin; Ur, terminal Urea; sCr, terminal Serum creatinine; eGFR, terminal Estimated glomerular filtration rate; Hemoglobin, terminal Hemoglobin; Urine protein, terminal Urine protein was present; AKI, Acute Kidney Injury; PCT, procalcitonin; Culture, terminal hematuria sputum culture status any one is positive; PRA, panel reactive antibodies; HLA mm, HLA mismatch; DGF: Delayed Graft Function; LOH: The length of post-kidney transplant hospitalization; ATG, Antihuman thymocyte globulin; TAC, Tacrolimus; CsA, Cyclosporin A; MMF, Mycophenolate mofetil; GC, Glucocorticoid.

### Analysis of risk factors for the evolution of BK viruria to BK viremia

A univariate analysis of BK viremia was performed, revealing variables with *p*-values below 0.1, including donor age, donor diabetes (yes), high BK viruria, recipient age, and immunoinduction regimen (ATG). Following inclusion of these variables in a multivariate analysis, it was determined that high BK viruria [OR: 11.641 (1.745, 77.678), *p* = 0.011], recipient age [OR: 1.106 (1.017, 1.202), *p* = 0.018], and immunoinduction regimen [ATG; OR: 0.063 (0.006, 0.683), *p* = 0.023] were independent predictors of BK viremia. Table 4 provides detailed data regarding the analysis.

**Table 4:** Univariate and multivariate analysis of BK viruria evolving into BK viremia.

Variable	Single-factor analysis, OR (95% CI)	*p*-value	Multiple-factor analysis, OR (95% CI)	*p*-value
Immunoinduction regimen(ATG)	0.083 (0.01,0.675)	0.083	0.063 (0.006,0.683)	0.023
Donor age, year	1.042 (0.993,1.094)	0.094		
Donor diabetes(yes)	4.107 (0.969,17.413)	0.055		
High BK viruria	7.241 (1.511,34.705)	0.013	11.641 (1.745,77.678)	0.011
Recipient age	1.077 (1.01,1.145)	0.018	1.106 (1.017,1.202)	0.018

OR, odds ratio; 95% CI, 95% confidence interval; ATG, Antihuman thymocyte globulin.

### Time distribution of BK virus infection in kidney transplant recipients

**Figure 2:**
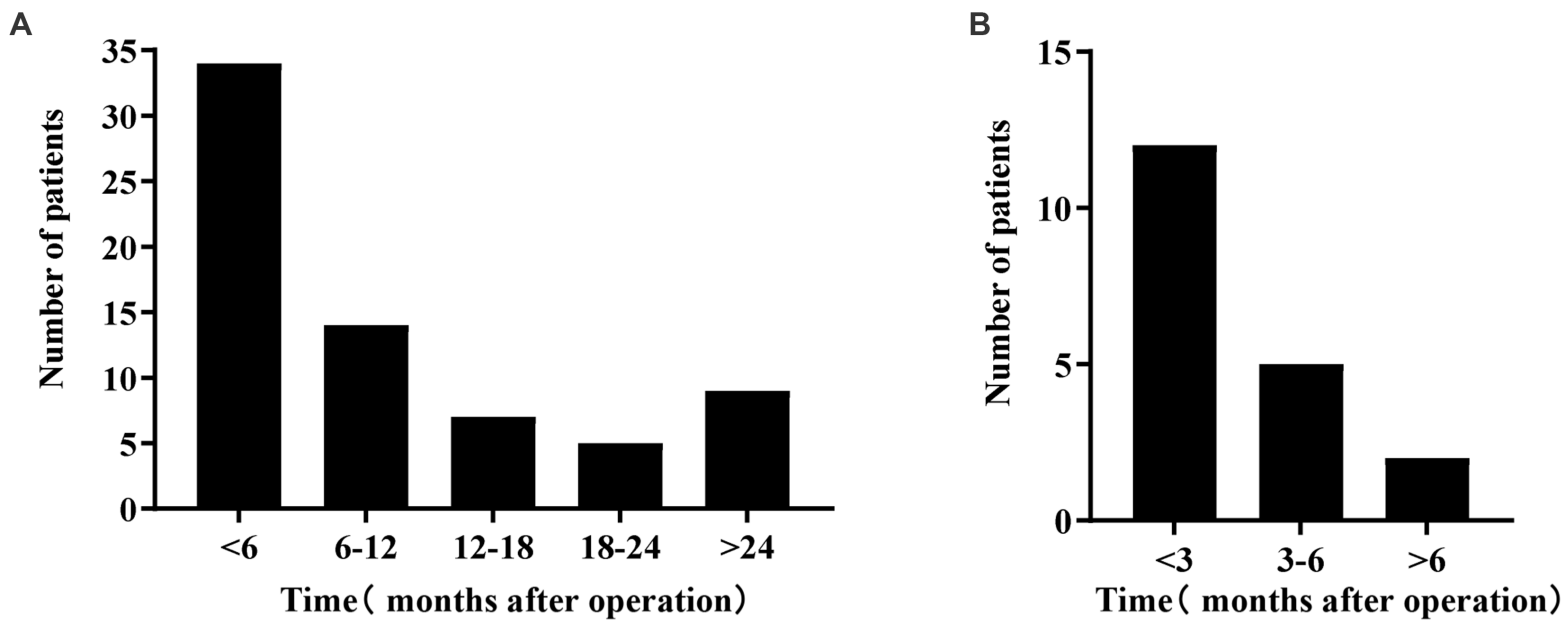
**(A)** Shows the time distribution of BK viruria after kidney transplantation. The horizontal axis represents the time after kidney transplantation, and the vertical axis represents the number of patients. This graph shows the highest incidence within 6 months of the transplant; it decreases over time and then increases again 2 years later. **(B)** Shows the time distribution of BK viremia after BK viruria. The horizontal axis shows the time after BK viruria, and the vertical axis shows the number of patients. This graph shows that BK viremia occurs mainly within 3 months of diagnosis of BK viruria.

The study revealed that BK viruria had an incidence of 15.6%, with a median follow-up period of 1,072 days (range 739–1,418). Figure 2A displays the time distribution of 69 kidney transplant recipients with BK viruria, with a median follow-up time of 185 days (range 82–387). The majority of BK viruria cases occurred within the first 6 months following kidney transplantation, accounting for nearly 49.3% of cases. Over time, the incidence of BK viruria decreased, but there was a rebound observed 2 years after surgery. Furthermore, the study reported a BK viremia incidence of 5.4%. BK viremia was discovered either simultaneously with or after BK viruria, and among kidney transplant patients with BK viremia, 63.2% were diagnosed within 3 months after BK viruria, with a median follow-up time of 26 days (range 0–119). Figure 2B illustrates the time interval between the diagnosis of BK viruria and BK viremia in 19 kidney transplant recipients.

### Application of machine learning in assessing the evolution of BK viruria to BK viremia

**Figure 3:**
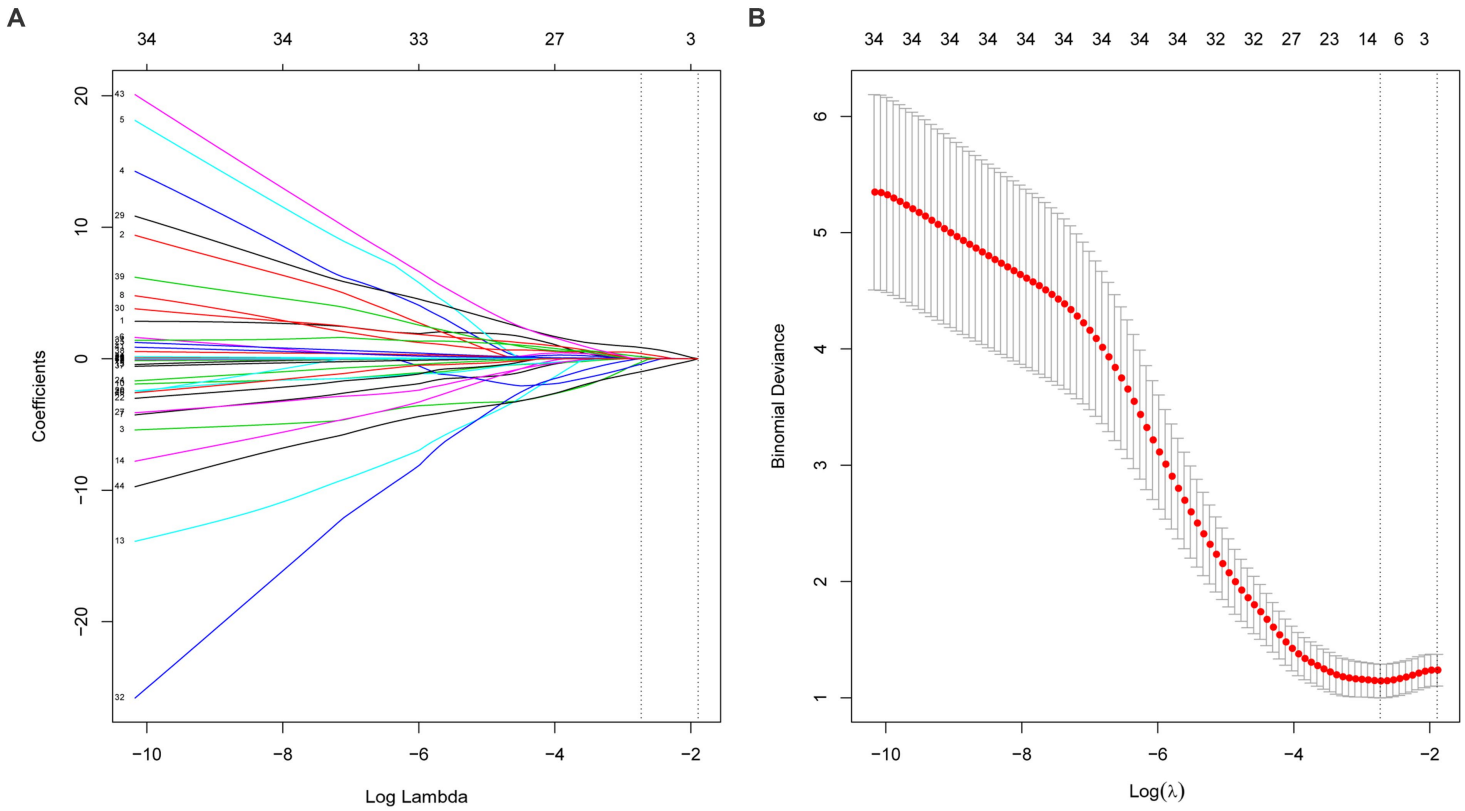
**(A,B)** Show the variable selection process of lasso regression. When the minimum mean square error of λ was 0.065, the variables were reduced to eight.

**Figure 4:**
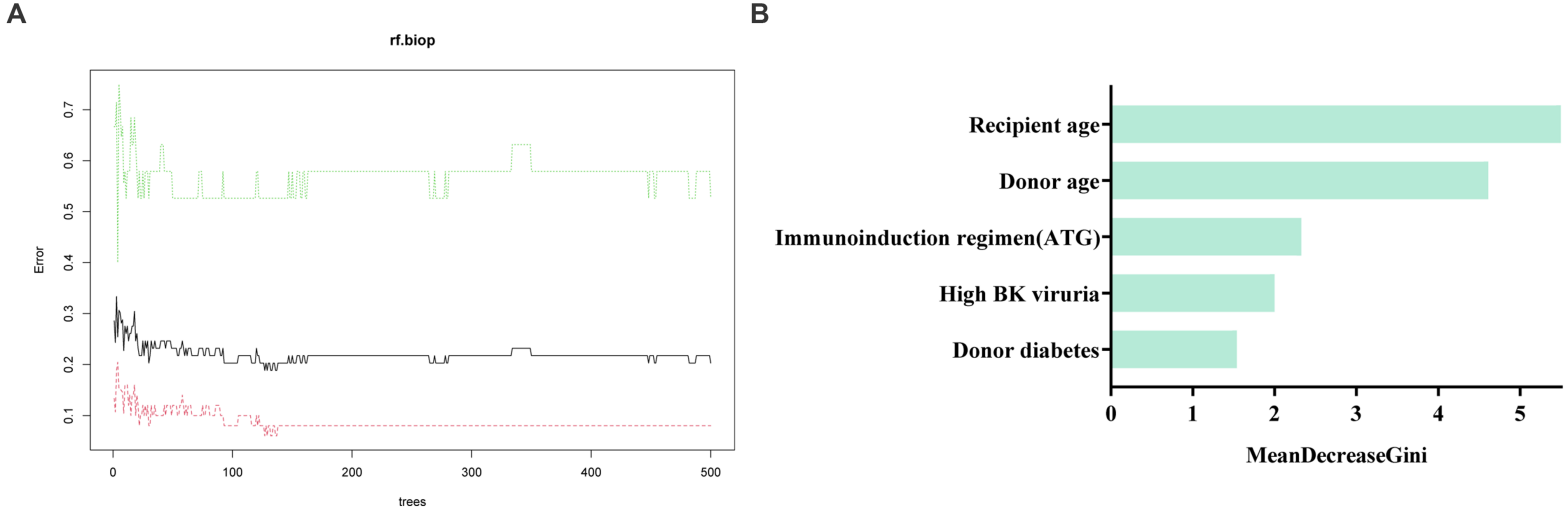
**(A)** shows the training process of the random forest model. The optimal number of trees of the random forest model is 127. **(B)** Displays the results of the Mean Decrease Gini importance analysis, which ranks the importance of features after random forest screening. The analysis identified recipient age, donor age, immunoinduction regimen (ATG), high BK viruria, and donor diabetes as the top five important factors in descending order.

We further used machine learning approaches to analyze the importance of potential risk factors for the evolution of BK viruria to BK viremia. We used a lasso regression approach for data dimensionality reduction, and when the minimum mean square error of λ was 0.065, the variables were reduced to eight, namely: recipient transplantation etiology (polycystic kidney disease), recipient blood group (O), recipient DGF, recipient age, high BK viruria, recipient immunoinduction regimen, donor diabetes and donor age. The variables were screened as shown in Figures 3A,B. Furthermore, we utilized the random forest model to identify the top five variables for predicting BK viremia, as illustrated in Figures 4A,B. It is noteworthy that the potential risk factors obtained by conventional logistic regression were among the top five variables.

## Discussion

BK virus is commonly present in the kidneys of most adults. However, in kidney transplant patients who receive immunosuppressive therapy for an extended period of time, the virus can become reactivated. The process starts with lysis of renal tubule cells, leading to urinary excretion of the BK virus. The virus then replicates in interstitial cells and penetrates the peritubular endothelial barrier to enter the bloodstream, resulting in BK viremia. Once the virus reaches the allograft, it attacks renal tubular epithelial cells, causing interstitial fibrosis and leading to the development of BKVN. This condition can ultimately result in renal graft degeneration and transplant failure. A study has shown that approximately 33% of patients with BK viruria progress to BK viremia and subsequently to BKVN without any intervention (7).

In this study conducted at a single center, 69 kidney transplant recipients developed BK viruria, of whom 19 also developed BK viremia. The rate of progression to BK viremia was 27.5%, which was slightly lower than the 33% reported in other studies. No BKVN was identified as no kidney biopsy was performed. Similar to findings from other centers, the incidence of BK viruria at our center was highest within the first 6 months post-transplantation, followed by a declining trend. However, there was a subsequent increase in incidence observed at the 2-year post-transplantation mark (21–23). The study findings indicate that the majority of BK viremia cases (63.2%) occurred within the first 3 months following BK viruria. As there are currently no specific antiviral therapies for BK virus-related diseases, kidney transplant patients typically rely on reducing immunosuppressant doses and changing immunotherapy regimens. Although this approach can increase the risk of chronic rejection, early detection and intervention of BK viruria and BK viremia are beneficial in reducing the incidence of BKVN.

Prior research has identified several potential risk factors associated with postoperative BK virus infection in kidney transplant patients, including recipient age, deceased donor, tacrolimus regimen and male recipient (6, 9, 24). While previous research has established that deceased donors are a risk factor for BK virus infection, few studies have examined the specific risk factors associated with DCD. In this study, all included kidney donors were DCD donors, and tacrolimus regimen is used by 97.7% of the population. Our analysis revealed that donor age may be an independent risk factor for BK viruria [OR: 1.022 (1.000, 1.045), *p* = 0.047]. Deceased donors are typically older, and in this study, the average donor age was 54.00 [45.00, 61.00] years. The percentage of ECD is as high as 42.8%. Notably, the age of donors in the control group was significantly lower than that in the BK viruria group (53.00 [44.00, 60.00] vs. 58.00 [47.00, 63.00], *p* = 0.01). Advanced donor age is often indicative of poor kidney quality, and it may be a contributing factor to BK virus infection. The biomarker PCT, which is linked to bacterial infection and inflammation, has been found to be a useful predictor of AKI in critically ill patients (25, 26). Our study revealed that the PCT range of 0.5 to 10 ng/ml before donation from deceased donors had a protective effect against BK viruria infection [OR: 0.482 (0.280, 0.828), *p* = 0.008], but not against the progression of BK viruria to BK viremia. Since DCD donors have longer Intensive Care Unit (ICU) stays, bacterial infections may still occur despite efforts to avoid sepsis. The PCT range of 0.5 to 10 ng/ml may reflect this phenomenon. Bacterial infections may activate the immune system, which could inhibit BK virus replication. Moreover, DCD donors often receive multiple antibiotics, and it is worth exploring whether the antiviral properties of these antibiotics inhibit the growth of microorganisms that promote BK virus transmission.

Following kidney transplantation, some patients may experience progression from BK viruria to BK viremia as a result of BK virus infection. The early identification of high-risk factors for developing BK viremia is of particular importance. Through traditional logistic regression analysis, we identified three independent risk factors, including recipient age [OR: 1.106 (1.017, 1.202), *p* = 0.018], high BK viruria [OR: 11.641 (1.745, 77.678), *p* = 0.011], and the immunoinduction regimen [ATG; OR: 0.063 (0.006, 0.683), *p* = 0.023]. Consistent with prior literature, recipient age and high BK viruria were found to be independent risk factors for the development of BK viremia (20, 27–31). For example, one study found that the BKPyV urine assay that best distinguished between positive and negative BK viremia was 6.71 log10 copies/ml [AUC = 0.953, *p* < 0.001 (30)]. Another single-center study from Thailand also reported the positive effect of urinary BK viral load in predicting BK viremia (31). In contrast to previous studies, our results suggest that the use of ATG as an immune induction method is protective against the progression of BK viruria to BK viremia, as compared to the use of basiliximab. Typically, ATG has a stronger immunosuppressive effect than basiliximab, which has a higher risk of infection. For instance, a clinical study of low-risk living kidney transplants found that immune induction with ATG was a risk factor for BK viremia infection (32). However, a different study came to a different conclusion, stating that the occurrence of BK viremia was not related to the mode of immunosuppression induction (33). To reduce the incidence of rejection after renal transplantation, our center routinely performs postoperative immune induction with basiliximab. However, for recipients who receive high-risk donor kidneys, we switch the immune induction method to ATG. Additionally, a tacrolimus-based triple suppression regimen is used for immunosuppressive maintenance in 97.7% of our population. The difference in the study population may also account for the variation in results, as all of our study participants received DCD donor kidneys, with 42.8% of them being ECD donors.

In addition, we used a machine learning approach to assess important variables associated with the progression of BK viruria to BK viremia. The results showed that recipient age, high BK viruria and immunoinduction regimen (ATG) appeared in the top five variables of the random forest model. Thus when patients present with BK viruria, close attention to such patients and timely intervention may be helpful in the progression of BK virus.

This study aims to identify risk factors associated with BK virus infection and the progression of BK viruria to BK viremia, using a machine learning approach to evaluate the significance of potential variables in predicting such progression. These factors are critical in our study population and can aid clinicians in making informed decisions. However, certain limitations must be considered. Firstly, in order to obtain complete clinical data, we excluded donor kidneys from other hospitals, which may slightly underestimate the actual incidence of BK virus infection. Secondly, the results of our study may not be generalizable to other centers, as all participants were recipients of DCD donor kidneys, close to half of which belonged to ECD, and the majority of recipients were receiving a tacrolimus-dominant triple immunosuppressant postoperatively. Therefore, the difference in the study population may affect the results. Thirdly, we acknowledge that there is a sample size problem in single-center studies, and therefore, further expansion of the sample size is needed to validate our findings.

## Conclusion

BK virus infection in kidney transplant recipients is influenced by multiple factors related to both the donor and the recipient, particularly in the context of DCD. Identifying and screening high-risk groups for BK virus infection and implementing early intervention and treatment can help prolong the lifespan of the transplanted organ.

## Data availability statement

The raw data supporting the conclusions of this article will be made available by the authors, without undue reservation.

## Ethics statement

The studies involving human participants were reviewed and approved by Ethics Committee of Renmin Hospital of Wuhan University. The patients/participants provided their written informed consent to participate in this study.

## Author contributions

JZ and TQ designed the study. YL and CK carried out data collection and analyzed the data. TW, YZ, and HH made the figures. YL, HH, and CK drafted and revised the paper. All authors contributed to the article and approved the submitted version.

## Funding

This study was supported by the National Natural Science Foundation of China (Reference numbers: 81870067 and 81400753).

## Conflict of interest

The authors declare that the research was conducted in the absence of any commercial or financial relationships that could be construed as a potential conflict of interest.

## Publisher’s note

All claims expressed in this article are solely those of the authors and do not necessarily represent those of their affiliated organizations, or those of the publisher, the editors and the reviewers. Any product that may be evaluated in this article, or claim that may be made by its manufacturer, is not guaranteed or endorsed by the publisher.
